# AVIDbase: A biologically accurate structural dataset of nanobody‐antigen complexes

**DOI:** 10.1002/pro.70773

**Published:** 2026-08-24

**Authors:** Tadej Medved, Jurij Lah, Goran Miličić, San Hadži

**Affiliations:** ^1^ Biologics Drug Product Development Novartis LLC Mengeš Slovenia; ^2^ Faculty of Chemistry and Chemical Technology University of Ljubljana Ljubljana Slovenia

**Keywords:** assembly, database, interface, nanobody, PTM

## Abstract

Accurate structural data in a standardized format is one of the key factors behind the success of machine learning (ML)‐based methods for protein design and structure prediction. However, their application to nanobody‐antigen complexes has lower success rates compared to globular protein complexes, partly due to the limited amount of high‐quality structural data. While several dedicated databases already exist, automated assembly pipelines frequently overlook various artifacts, which act as additional noise and limit the effectiveness of ML applications. Common issues include incorrectly defined antigen assemblies, redundancy bias, inclusion of crystal contacts, strained geometry due to crystal packing, as well as missing density or post‐translational modifications near the interface. To address these issues, we present **A**ntigen‐**V**HH **I**nterface **D**atabase (**AVIDbase**), a highly curated dataset of nanobody‐antigen structures. In addition to correcting structural artifacts, the dataset provides a nonredundant set of structures with standardized chain identifiers, harmonized metadata, and cleaned atomic coordinates in a ready‐to‐use format for ML applications. AVIDbase is available on GitHub (github.com/Novartis/AVIDbase) and Zenodo (doi.org/10.5281/zenodo.20488703).

## 1 INTRODUCTION

Nanobodies, also known as VHH, are single‐domain, heavy chain‐only antibody fragments derived from camelids. Increased solubility, small size, and monomeric format make nanobodies an easier‐to‐produce alternative to conventional antibodies while exhibiting similar antigen‐binding affinities (Jin et al., 2023). Structural analysis of larger datasets revealed that, compared to conventional antibodies, nanobodies can recognize more buried and concave epitopes, also by employing framework residues for antigen binding (Gordon et al., 2023; Zavrtanik et al., 2018). Nanobodies are also capable of recognizing more exotic antigens such as *de novo* designed proteins made of coiled coils (Majerle et al., 2021) and low‐immunogenic DNA structures such as G‐quadruplexes (Galli et al., 2022; Pevec et al., 2025).

VHH binders are typically obtained by screening natural or synthetic immune libraries with multiple rounds of selection via methods such as phage display (Jin et al., 2023). This approach is experimentally intensive, which motivated the development of *in silico* screening and even *de novo* nanobody design approaches, with recent *in silico* diffusion‐based methods such as RFDiffusion (Bennett et al., 2025) or BoltzGen (Stark et al., 2025) reporting initial success. Other ML‐based applications for VHH include prediction of the Δ*G* of binding (El Salamouni et al., 2025; Feng et al., 2024) and VHH‐antigen complex prediction with models such as AlphaFold3 (Abramson et al., 2024) and Boltz‐2 (Passaro et al., 2025) for *in silico* screening. However, cutting‐edge predictors for both affinity and binding pose are still suboptimal for antibody–antigen complexes, due in part to high flexibility and weak coevolutionary signals in the CDR loops (Yin & Pierce, 2024).

The success of in silico approaches incorporating VHH‐antigen complexes depends on prior data quality, underscoring the need for datasets addressing both redundancy and experimental bias. Ideally, such datasets should have a standardized format (relevant for VHH numbering schemes), be devoid of structural artifacts, and closely recapitulate the VHH‐antigen interface as formed in solution. This issue is particularly relevant for VHH‐antigen structures obtained by x‐ray crystallography, which can introduce data bias as a result of missing electron density, distortions in interface geometry due to crystal packing or non‐biological assemblies within the asymmetric crystallographic unit. Such artifacts are known to result in lower correlation with experimentally measured affinity (Nivón et al., 2013; Shringari et al., 2020). Much work has already been done to compile and standardize existing antibody and VHH structural data for downstream applications. The AbDb database (Ferdous & Martin, 2018) and the Zavrtanik dataset (Zavrtanik & Hadži, 2019) were the first to compile standardized VHH interface structures. The most widely used antibody database currently is OPIG's SAbDab (Dunbar et al., 2014), with a user‐friendly interface, weekly updates from the PDB, and database filtering functionality. However, SAbDab's shortcomings such as a lack of fully standardized structures and improperly defined biological assemblies necessitate additional processing steps to prepare the structures for ML input. These problems stem from the incompleteness of the raw PDB data, wherein macromolecule names, source organisms, and biologically relevant assemblies can be incorrectly assigned, which acts as additional noise in downstream applications. This has motivated the creation of newer databases incorporating automated pipelines that address these concerns in different ways. ANDD (Wu et al., 2026) integrates sequence, structure, and affinity data from SAbDab, the PDB, and other databases into a ready‐to‐use dataset for fine‐tuning of generative models, but does not perform any additional processing of PDB structures. AbSet (Almeida et al., 2025) takes steps to standardize the experimental structures, automatically determining correct VH‐VL and antibody–antigen complexes, and supplementing them with an in silico dataset of predicted structures. SAAINT‐DB (Huang et al., 2025) similarly standardizes PDB structures and provides extensive metadata for each biological interface, while also building missing sidechains to ensure completeness. ANABAG (Grandguillaume et al., 2025) uniquely considers explicit glycosylation patterns and different levels of sequence‐ and structure‐based redundancy, while removing crystallization artifacts via energy minimization. However, it only considers SAbDab‐defined biological assemblies, potentially including incorrect or incomplete biological interfaces. The most recent dataset, SNAC‐DB (Gupta et al., 2026), goes a step further and explicitly separates biological interfaces and crystal contacts with a manual review‐supplemented automatic pipeline, making both subsets available in a plug‐and‐play format for ML applications.

Despite these improvements in VHH‐antigen structural data curation, none of the above approaches provides a fully integrated dataset that is corrected for all structural artifacts and redundancy. Structures are not filtered based on interface missing density, biological assemblies can be incorrectly assigned, and heteroatomic species including post‐translational modifications (glycosylation, phosphorylation), noncanonical amino acids, and obligate cofactors are all disregarded, potentially causing information loss for structure or affinity prediction. To address these gaps, we present **A**ntigen‐**V**HH **I**nterface **D**atabase (**AVIDbase**), which seeks to preserve the full biological context of VHH‐antigen interfaces while remaining easily accessible via extensive metadata annotation and harmonization. We combine the best practices observed in existing databases with explicit consideration of post‐translational modifications, cofactors, and missing density, while employing structure minimization and repacking to remove crystal artifacts in order to maximize the quality of the existing data both for structural bioinformatics and ML applications.

## 2 RESULTS

### 2.1 Correction of biological assembly artifacts and improvement of interface fidelity

**FIGURE 1:**
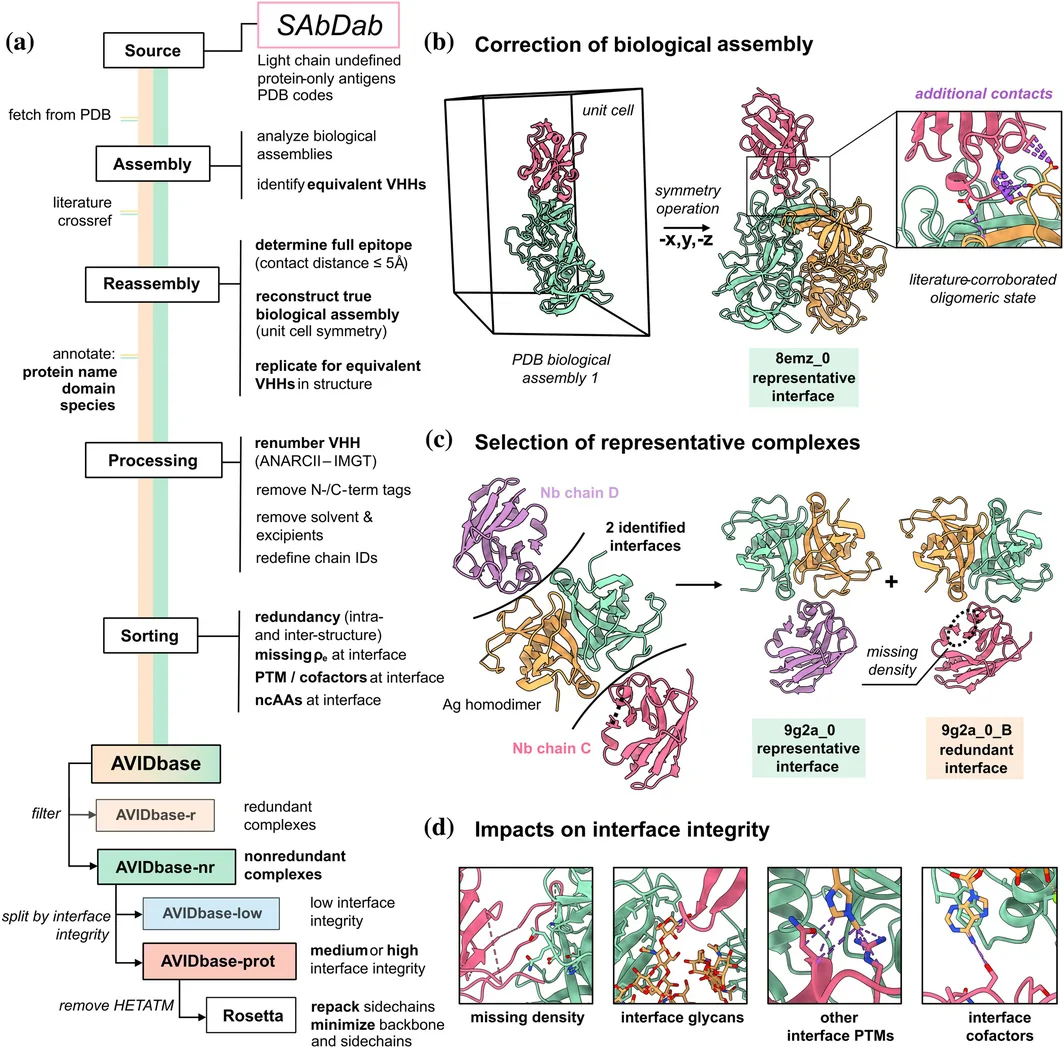
AVIDbase preparation pipeline. (a) Outline of the dataset preparation pipeline. Structures were sourced from the PDB based on SAbDab annotations, relevant biological assemblies were extracted and modified to recapitulate the biologically most informative interface. All structures were preprocessed by removing crystallographic and sequence artifacts, and chain IDs and sequences were standardized. The resulting dataset was split into redundant (AVIDbase‐r) and nonredundant (AVIDbase‐nr) complexes, and the latter was further sliced based on interface integrity—missing density, PTMs and cofactors near the interface—into the protein‐only interaction dataset AVIDbase‐prot, which was further repaired with Rosetta. (b) Example of manual assembly reconstruction to preserve biological context (PDB: 8emz). Literature data reports antigen VP1 as homodimeric while deposited biological assembly is monomeric. Reconstruction of homodimer reveals additional contacts between the VHH and the second VP1 monomer. (c) Example of determining redundancy within a single structure (PDB: 9g2a). The biological assembly consists of 2 equivalent VHH binding the same epitope on symmetric sides of the antigen—the more complete interface is classified as representative, and the second interface with missing density as redundant. (d) Overview of structural artifacts and heteroatomic species affecting protein–protein interface integrity. Nanobody structures are colored in pink, antigen structures in mint, and the focused species in orange.

AVIDbase was prepared by combining automatic processing and extensive manual curation to ensure comparability, biological fidelity, and ease‐of‐use. The dataset preparation workflow is shown in Figure 1a, and the full structure of the dataset is outlined in Supplementary Table S1. Briefly, all x‐ray crystal structures in SAbDab containing single heavy chain antibodies interacting with at least one other protein (890) were retrieved from the PDB. The cutoff date for released structures was February 9, 2026. Structures with small disordered peptide antigens (19) were discarded, as well as the structures containing VNAR (7), megabody (1), Fab (4), or TCR heavy chains (1), and multispecific VHH formats (6) unless they acted as antigen (2). Antigen name and source organism annotations were improved and standardized throughout the dataset, as described in the Supplementary Text.

Next, structures were subjected to semi‐automated review to identify all non‐crystallographic VHH‐antigen interfaces. In several cases, neither the PDB asymmetric unit nor annotated biological assemblies corresponded to the correct assembly, which was then built using the appropriate symmetry operation defining the unit cell. For example, structure 8emz, as deposited in the PDB, lacks half of the interface from the second chain of the VP1 P‐domain homodimer, which can be obtained using symmetry operation (Figure 1b). In total, 1% (8) of unique AVIDbase complexes were constructed from symmetry mates, 61% (422) were obtained from the asymmetric unit, and 38% (258) from the existing biological assemblies as annotated in the PDB. Thus, each entry represents an interface record of (depositing author‐defined) chain IDs comprising the true biological assembly—a complex between a single VHH and its antigen in a minimum required oligomeric state (including both obligate homooligomers and biological heteromeric protein–protein interactions).

To address the redundancy of identical interfaces (either within one multichain PDB or across several PDBs of the same complex) we selected a single, most complete and highest‐resolution interface as the representative. For example, for structure 9g2a with two equivalent interfaces, the representative interface is assigned a unique ID 9g2a_0, while the redundant interface is assigned 9g2a_0_B (Figure 1c). Thus, each VHH‐antigen interface has a unique identifier based on the PDB code and an incremented interface index, with redundant interfaces distinguished by variant labels.

Additionally, structures were standardized and cleaned of crystallization artifacts. All VHH molecules were set chain ID “H” and renumbered according to the IMGT scheme. Solvent molecules and expression tags were removed, while biologically relevant components such as glycans, cofactors, and noncanonical residues were retained but with reassigned chain IDs as described in Methods and Supplementary Table S2. The complete AVIDbase dataset contains 1741 recorded VHH‐antigen interfaces, with 690 nonredundant interfaces in the subset AVIDbase‐nr, comprised of 672 unique VHH sequences and 342 unique antigens at the source organism level.

Finally, all complexes were annotated based on interface integrity (Methods). Structures with issues such as missing density, glycan molecules, noncanonical residues, or cofactors near the VHH‐antigen interface were classified as “low integrity” (Figure 1d). Among 690 unique interfaces, 7% (48) have missing density within 7 Å of the interface, 4% (30) have sub‐5 Å contacts with glycans, and 1% (7) with cofactors, and 0.4% (3) have noncanonical amino acids in the interface. A 3.5 Å resolution cutoff was applied to the remaining medium‐to‐high integrity structures, which resulted in the ML‐ready AVIDbase‐prot subset (Figure 1a). AVIDbase‐prot consists of 591 nonredundant nanobody‐antigen complexes with correct biological assembly in a standardized and cleaned data format, without missing density or non‐protein contacts near the interface. Additionally, the filtering of low‐integrity and low‐resolution removes outlier structures with high temperature B‐factors at the interface while preserving the distribution of interface area (Supplementary Figure S1). AVIDbase‐prot may be further filtered via the missing density‐related columns outlined in Supplementary Table S1 to obtain a more stringent subset of structures with complete protein chains only.

### 2.2 Structure repacking and minimization improves strained interface geometry due to crystal packing

**FIGURE 2:**
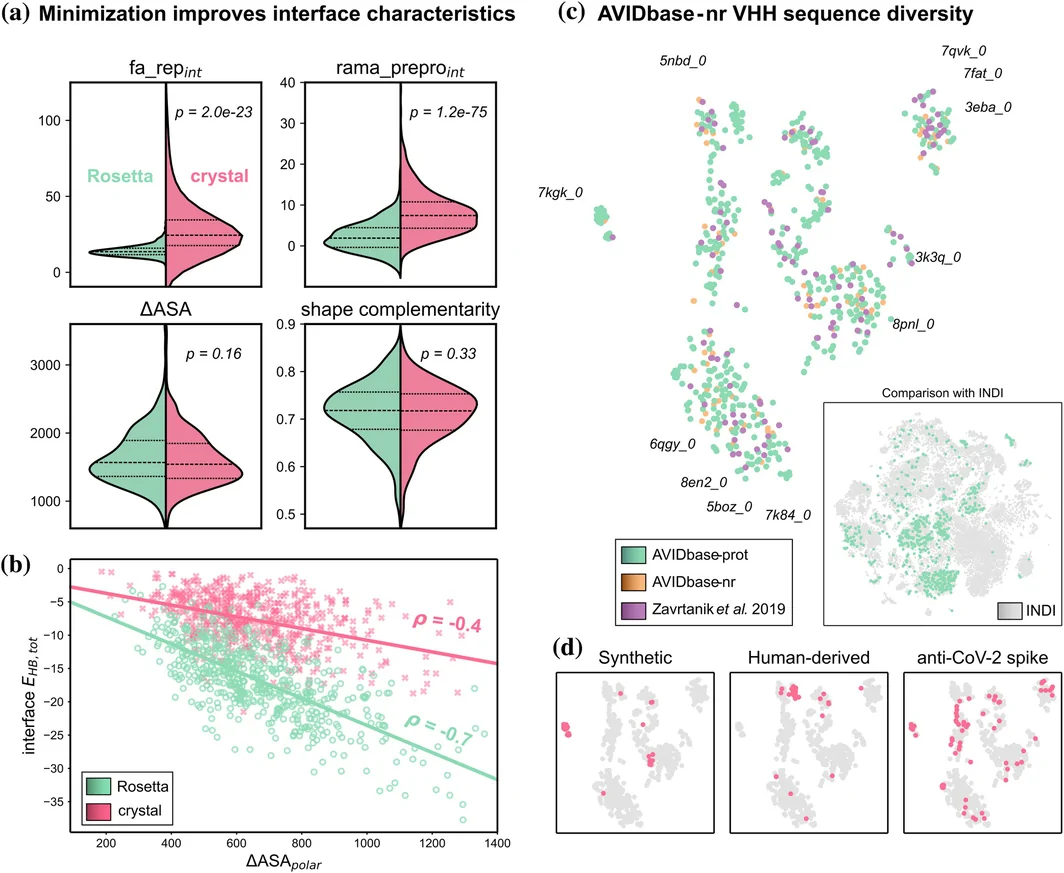
General characteristics of AVIDbase‐nr. (a) Violin plots comparing select descriptor distributions for AVIDbase‐prot before (red, right) and after repacking and minimization with Rosetta (green, left). Reported *p*‐values were calculated via Welch *t*‐test and corrected for multiple hypothesis testing with the Benjamini–Hochberg procedure at *α* = 0.05. (b) Scatter plot comparing Spearman correlations between the interface buried polar ASA and the total computed H‐bond energy in Rosetta before (red) and after repacking and minimization (green). Linear regression curves are shown as solid lines. (c) Comparison of VHH sequence diversity between AVIDbase and the Zavrtanik nonredundant nanobody dataset (Zavrtanik & Hadži, 2019). Shown is a 2D t‐SNE projection of nanoBERT (Hadsund et al., 2024) sequence embeddings for AVIDbase‐prot (green), AVIDbase‐nr (orange) and the Zavrtanik set (purple), with convex hull points annotated by their AVIDbase unique ID. A separate t‐SNE projection was calculated to compare AVIDbase‐prot (green) and a subset (38,037 sequences) of the nanobody sequence database, INDI (Deszyński et al., 2022) (gray). (d) Highlighted subsets (pink) of synthetic nanobodies, human‐derived VH‐only molecules and anti‐SARS‐CoV‐2 spike RBD nanobodies in AVIDbase‐nr embedding space (gray).

To achieve better representation of the VHH‐antigen interface as found in solution rather than crystal, we repacked and minimized the AVIDbase‐prot structures and filled the missing sidechains using Rosetta (Alford et al., 2017). Comparison of Rosetta score terms summed across interface residues between the original crystal and minimized structures shows that repacking and minimization significantly lowers the all‐atom vdW repulsive energy term among interface residues (fa_rep_int_) as well as interfacial backbone torsional energies (rama_prepro_int_) (Figure 2a). These changes represent fine‐grained optimization of individual residues that do not lead to large‐scale changes of the interface, as evidenced by similar distributions of interface buried surface area (ΔASA) and shape complementarity in both minimized structures and the original crystal (Figure 2a). Additional properties such as the interface electrostatic Rosetta energy terms are improved after minimization, with fewer buried unsatisfied polar atoms in the bound structure and a lower total Rosetta score on the nanobody side. Conversely, the interface all‐atom vdW attractive energy term, number of interface residues, and contact molecular surface—a measure of interface packing (Cao et al., 2022)—are not significantly altered after repacking and minimization (Supplementary Figure S2). Minimized structures also show significantly improved correlation between the buried polar interface area and the sum interface H‐bond energy (Figure 2b). This indicates that Rosetta repacking and minimization removes steric clashes and geometric strain induced by crystal packing, resulting in a more physically realistic model of the VHH‐antigen interface.

### 2.3 Representation of nanobody sequence space diversity

To assess how representative the dataset is in terms of nanobody sequence diversity, we used representations derived from the nanobody‐specific language model nanoBERT (Hadsund et al., 2024). A t‐SNE projection of these representations shows that AVIDbase‐prot covers all clusters in the nonredundant sequence space, meaning that little sequence diversity is lost when removing “low‐integrity” or low‐resolution complexes. Interestingly, a comparison with the early Zavrtanik nanobody set (Zavrtanik & Hadži, 2019) shows that the original 123 complexes already captured the majority of crystallizable VHH sequence space, and that the fivefold increase in structural data mainly improves sampling of the local embedding space. AVIDbase‐prot embeddings were also projected into the embedding space of a subset of the nanobody sequence database INDI (Deszyński et al., 2022), comprised of 20,000 randomly sampled NGS sequences and 18,037 sequences from other sources. This projection shows that large regions of nanobody sequence space are still structurally unexplored and point to areas in sequence space with uncharacterized antigen recognition mechanisms (Figure 2c).

The bulk of recorded nonredundant VHH molecules consists of traditional nanobodies sourced from *Camelidae* species (626); however, a non‐negligible number of synthetic nanobodies (35) and human single‐domain antibody fragments (27) are present. These molecules form small clusters in AVIDbase‐nr sequence space (Figure 2d), additionally with individual molecules present in primarily *Camelidae*‐populated clusters. Embedding space is also useful for considering VHH diversity against a single antigen; for example, the VHH against SARS‐CoV‐2 RBD are markedly varied and appear in each of the observed clusters (Figure 2d), highlighting how immune libraries across multiple campaigns are better able to sample both germline and somatic mutational space.

### 2.4 Addressing antigen bias through identification of unique epitopes by clustering

**FIGURE 3:**
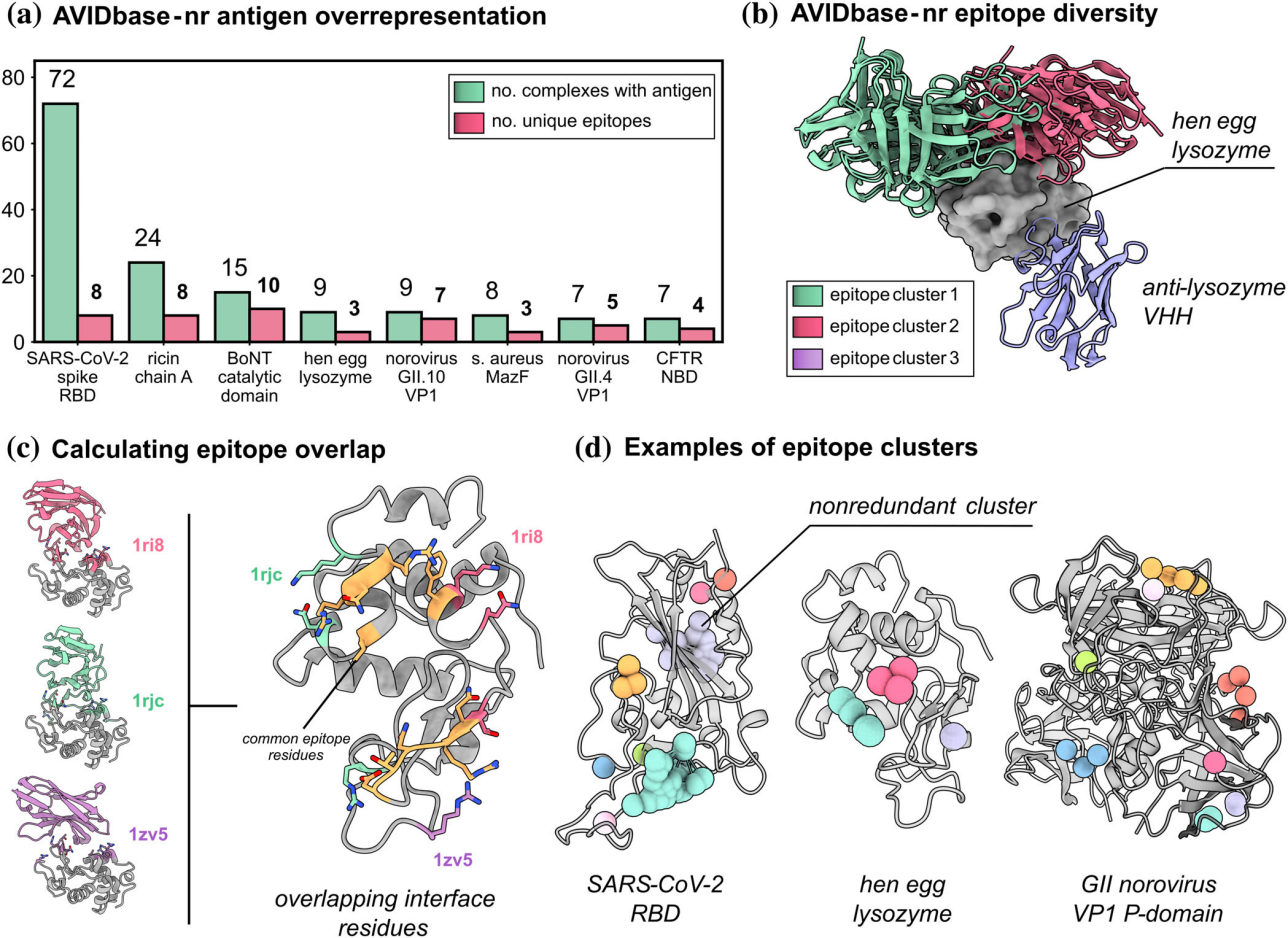
Analysis of epitope‐level redundancy in AVIDbase‐nr. (a) Bar plot of the most overrepresented antigens in AVIDbase‐nr (total count in green, number of unique epitope clusters in red). (b) Example alignment of all VHH‐hen egg lysozyme complexes against a reference antigen structure (gray). VHH are colored in mint, pink or purple. (c) Example of a common epitope between VHH‐lysozyme structures 1ri8 (pink), 1rjc (mint) and 1zv5 (purple). Lysozyme is shown in gray, the overlapping interface residues in orange, and the non‐overlapping residues are colored by their corresponding structure. (d) Depiction of epitope clusters on 3 separate antigen structures—SARS‐CoV‐2 spike RBD, hen egg lysozyme and the GII norovirus VP1 P‐domain. Individual interfaces are represented by their C_*α*_‐centroids as spheres colored based on their corresponding cluster and connected by lines of the same color.

A large number of nonredundant nanobodies (151 complexes, 22%) bind only to a fraction of overrepresented antigens (Figure 3a). These typically belong to therapeutic targets (Lam et al., 2022; Naidoo & Chuturgoon, 2023; Rudolph et al., 2020) such as SARS‐CoV‐2, human norovirus, botulinum neurotoxin and ricin, or model proteins such as lysozyme. For example, over 10% of AVIDbase‐nr structures are VHH complexes with SARS‐CoV‐2 RBD (Figure 3a). Although these nanobodies have different sequences, structural inspection reveals that many of them bind to structurally identical epitopes. To explicitly consider redundancy at the epitope level and obtain a set of truly structurally unique VHH‐antigen interfaces, we used a custom epitope clustering approach. Briefly, overrepresented antigens were analyzed by aligning all AVIDbase‐nr complexes containing the same antigen (on the level of protein and domain) from the same species against a representative structure (Figure 3b, Methods). Unique epitope clusters were defined as groups of structures sharing more than 50% identical interacting residues (Figure 3c). With this approach, the previous 151 overrepresented complexes can be reduced to just 48 epitopes with minimal overlap in interacting antigen residues.

The results of this epitope clustering are demonstrated on three overrepresented antigens (Figure 3d). For the SARS‐CoV‐2 RBD, 8 unique epitopes were identified in 72 representative complexes. The main two clusters are an epitope at the ACE2 binding site (43 complexes) and a distal epitope near the *β*2‐strand, targeted to disrupt spike trimerization (21 complexes) (Huo et al., 2021). The third epitope recognized by multiple nanobodies lies near the *β*1‐strand (3 complexes) opposite the *β*2‐epitope, while the remaining 5 unique epitopes correspond to singular VHH molecules binding rare sites (7x2l_0, 7d2z_0) or regions flanking the ACE2 binding site (8h5t_0, 8zrd_0, 8owv_1).

The second example is hen egg lysozyme, a model protein for nanobody‐antigen interactions (De Genst et al., 2006). Out of 9 VHH‐lysozyme complexes, 3 unique epitopes were identified. The largest two epitope clusters are comprised of nanobodies binding two separate ends of the substrate binding site, split as in the literature (De Genst et al., 2006). The third epitope, corresponding to structure 1zvh_0, was likewise correctly assigned, as the corresponding VHH binds a short α‐helix away from the substrate binding site.

For the VP1 P‐domain from human norovirus genogroup GII (Kher et al., 2023), eight unique epitopes were identified in 19 complexes across 3 separate genotypes. Three main clusters were identified at the top of the P2‐subdomain dimer (4 complexes), and on either side of the P1‐subdomain interface (4 and 3 complexes respectively). 8emy_0 was assigned a unique cluster similarly as in the publication; however, 8en6_0 was in our case differentiated from the main P2‐subdomain cluster. Three separate clusters were identified on the bottom of the P1‐subdomain, split from the singular cluster as defined in the literature (Kher et al., 2023).

## 3 DISCUSSION

VHH‐antigen interface databases each possess their own benefits and drawbacks. For comparison, we considered subsets of the databases encompassing VHH‐antigen complexes from structures determined by x‐ray crystallography. Compared to SAbDab, AVIDbase contains 92% of SAbDab‐listed VHH‐antigen complexes, with the remaining 8% removed due to either incorrectly assigned antibody format or unstructured peptide antigen. Additionally, 42% of antigen oligomeric assemblies in AVIDbase differed from their corresponding definitions in SAbDab, with 20% of all AVIDbase entries and 14% of nonredundant entries capturing additional interface residues not considered in SAbDab structures. AbSet (Almeida et al., 2025) contains most of the original PDB structures used also in our work and takes steps both to standardize the numbering of its VHH molecules and remove structures with missing density; however, its workflow for assigning biological interfaces still retains some crystal contacts or non‐biological antigen assemblies (for example AbSet structures 2x89_2, 3ogo_2) and lacks searchable metadata. SAAINT‐DB (Huang et al., 2025) has a similarly complex parsing protocol and a data table annotating species and antigen names, while also performing reconstruction and repacking of unresolved atoms. SAAINT‐DB also contains 40 additional structures that were not found in SAbDab and are also absent from our work. However, it does not explicitly define redundancy and contains assignations of crystal contacts as interfaces (1bzq) or incomplete interfaces (5m2j, 5mje, 9g4g). For AVIDbase‐nr, 25% (170) of complexes have differently assigned biological assemblies compared to equivalent complexes in SAAINT‐DB, with 11% (78) capturing additional contacts. Similarly to SAAINT‐DB, ANABAG (Grandguillaume et al., 2025) contains incomplete biological assemblies, with 27% (187) of VHH‐antigen complexes assigned in a different oligomeric state than their AVIDbase‐nr equivalents. Additionally, 16% (108) of AVIDbase‐nr structures identify residue‐level contacts with antigen units that are discarded in ANABAG. The latest dataset, SNAC‐DB (Gupta et al., 2026), combines an automated pipeline with manual curation similarly to our approach, providing standardized structures with rebuilt unresolved residues, while explicitly classifying crystal contacts along with biological interfaces and considering structure‐based redundancy at different thresholds. Assignation of biological interfaces differs from AVIDbase‐nr in 3% of cases (27), primarily in complexes with non‐CDR contacts (4grw) or a small number of contacts with other chains (5m2j, 7ar0).

All of the above datasets were compiled with the intention of providing consistent, harmonized data for ML applications while maximizing the information gained from existing experimental structures. Thus, they incorporate extensible automated workflows that accurately assign biological interfaces in a majority of cases. However, the edge cases where misclassification arises can significantly impact the predictive power of a model, especially when predicting the binding affinity. These edge cases are difficult to resolve with fully automated approaches, as PDB‐deposited structures may contain incorrect or incomplete biological assemblies or metadata. AVIDbase addresses these cases via manual review of the corresponding literature, using experimentally determined oligomeric states to extract the true VHH‐antigen complex—in necessary cases building it directly from the crystallographic symmetry mates, which is not covered in the other databases. Uniquely, AVIDbase also explicitly considers glycans, noncanonical residues and bound cofactors in the structure, to properly record cases where non‐protein contacts contribute significantly to the interface. As missing residues can also impact predictive model quality, they are annotated as well, which has to our knowledge only been done in AbSet. This adds an additional layer of information useful for subsampling more complete interfaces to improve input data quality, as is represented by AVIDbase‐prot.

At the base level, AVIDbase considers redundancy based on CDR sequence, antigen name classification and source organism. This ensures that crystal structures with point mutations at the interface are still considered unique, due to the potentially disproportionate impact these mutations may have on the binding affinity, such as in the case of single‐point variants 1zvy_0 and 6jb2_0 (Akiba et al., 2019). Harmonization of both antigen name and species annotations also enables the user to apply custom filters at any desired level. We also cluster interface entries based on overlapping interacting residues, grouping complexes with varied binding poses against a common epitope. This could prove useful for cases where subsampling of interfaces with structurally distinct epitopes is required to reduce bias. To our knowledge, the only other two datasets to consider this are ANABAG and SNAC‐DB. Similarly to ANABAG, AVIDbase‐prot supplies Rosetta‐minimized structures, which provide a better starting point for training affinity predictors, as they improve the initial crystal structure's correlation with specific interaction energies (such as H‐bonds), and this approach is accepted in the literature to increase correlation with experimental binding affinity and mutational ΔΔ*G* (Nivón et al., 2013; Shringari et al., 2020). Nevertheless, repacking in Rosetta is inherently stochastic, and may, in some cases, lead to a departure from the ensemble of true residue configuration, both at the interface and across the full structure. Hence, unminimized versions of AVIDbase‐prot structures are still made available for use cases prioritizing higher fidelity to the original crystal structures.

AVIDbase's hybrid approach seeks to represent, as holistically as possible, currently available VHH‐antigen interface structural information by directly addressing edge cases of incomplete or incorrectly assigned biological interfaces. The end goal is to strike a balance in data relevancy for both structural biology and ML applications by providing an easily searchable and rigorously annotated dataset along with standardized PDB structures. The AVIDbase dataset and the accompanying source code are made available on Zenodo (doi.org/10.5281/zenodo.20488703) and GitHub (github.com/Novartis/AVIDbase), respectively. To ensure continued relevancy of the data, we plan to maintain both the code and the database, with periodic updates as newer nanobody‐antigen structures are deposited to the PDB.

## 4 METHODS

### 4.1 Dataset annotation

Metadata including molecule names, associated publications, UniProt accession codes, source organism species and subspecies corresponding to each polypeptide chain were extracted from the header of each downloaded mmCIF file. Protein sequences were similarly extracted and scored with ANARCII‐*accuracy* (Greenshields‐Watson et al., 2025), then all sequences classified as antibody heavy chains with a sequence score ≥ 24 were annotated as putative VHH molecules, and the rest as antigen sequences. Any misclassified formats such as Fabs, TCRs, or megabodies were manually removed. The biological relevancy of interfaces was assessed based on the number of atom‐atom contacts between CDR residues and antigen molecules in the structure (heavy atom pair distance ≤5 Å), as well as the annotated quaternary structure of the antigen as obtained from either the associated publication or UniProt. All necessary cofactors were likewise determined manually based on literature and UniProt annotations. The annotation of source organisms, protein names and subunits or domains was manually harmonized for comparability between equivalent entities, with a dictionary of changes provided in the source code.

### 4.2 Structure preparation

Biologically relevant complexes were built from each AVIDbase VHH‐antigen interface record in Python using the Biotite 1.6.0 (Kunzmann & Hamacher, 2018) and Biopython 1.86 (Cock et al., 2009) libraries. In brief, the chains specified in each interface record were loaded from either the specified biological assembly, asymmetric unit or symmetry mates. When building from symmetry mates, first the unit cell was generated and repeated once in each direction. Then, iterating over each chain ID specified in the interface record, the monomer with the highest ΔASA among units within 5 Å of that chain was retained until the desired antigen homooligomeric state was obtained. In all cases, each polypeptide chain was assigned a “symmetry index” starting at 0 and incremented by 1 for each duplicate chain ID in the structure, so that each monomer had a unique chain ID‐symmetry index combination. Residues with one or more alternate locations were filtered based on occupancy, with the first alternate location taking precedence in the case of a tie. All hydrogen atoms and solvent molecules were removed, along with any non‐glycan small molecules not in the interface record's list of expected cofactors. Cofactors and glycans without a bond to at least one protein residue were also removed. Any selenomethionines were changed into methionines by in‐place conversion of Se‐atoms to S‐atoms. All polypeptide chains in the structure were additionally preprocessed by removing residues missing at least one of their backbone N‐, C_*α*_‐ or C‐atoms. The VHH monomer was renumbered with ANARCII under the IMGT numbering scheme, and its CDR1‐3 sequences were extracted under a modified IMGT definition (Zavrtanik et al., 2018). N‐ and C‐terminal expression tag residues were removed, for the VHH according to its ANARCII numbering and for the antigen based on the *struct_ref_seq_dif* record in the mmCIF header. Finally, unique single‐letter chain IDs were assigned. The interacting VHH was assigned the “H” ID, while the antigen units, sorted by their unique chain ID‐symmetry index identifiers, were assigned uppercase IDs in alphabetical order. Glycans were assigned lowercase IDs corresponding to their covalently bound polypeptide chain, with identical residue numbers assigned to all monosaccharide units comprising a single bound glycan, differentiated by alphabetical insertion codes. Cofactors received the “X” ID and were assigned linear numbering. The resulting structures were exported to PDB files containing exclusively ATOM and HETATM rows.

### 4.3 Redundancy processing and interface integrity

Interface residues were determined to be those with at least one heavy‐atom contact (distance ≤5 Å) between 2 opposite residues, each with ASA ≥50 Å^2^ in the unbound state (Zavrtanik et al., 2018). Missing density spans were determined as all discontinuous backbone segments on a single chain, where discontinuity is defined as any backbone atom with a distance <1.2 Å or >1.8 Å from its preceding backbone atom. The missing density distance was computed as the shortest Euclidean distance of the C_*α*_‐atoms of the flanking residues from any heavy atom belonging to the binding partner on the opposite side of the interface. A similar scheme was adopted for the glycans and cofactors, except all glycan/cofactor heavy atoms were included in the distance calculation. Noncanonical interface residues were classified based on their presence in the interface residue set. Interface redundancy within a single structure was determined by ranking equivalent interface records based on closest missing density distance from the interface in descending order, followed by closest glycan and cofactor interface distances. Redundant interfaces across multiple structures were determined as those with identical CDR1‐3 sequence, antigen name and source organism. These were sorted by ascending crystallographic resolution, and the first interface with a missing density interface distance >7 Å was added to the nonredundant set if available; otherwise, the highest‐resolution structure was chosen.

The interface integrity was determined as follows: structures with 1 or more interface noncanonical residues, missing density interface distance <7 Å or at least 1 interface glycan at <4 Å were classified as “low integrity”; structures with a missing density <9 Å or an interface glycan/cofactor distance <5 Å as “medium‐integrity”; the rest were classified as “high‐integrity”.

### 4.4 Epitope clustering

Epitope redundancy was determined by comparing nonredundant VHH sequences binding to equivalent antigens from the same source organism. Sorting by resolution and filtering by interface integrity classification, the top‐ranked structure was chosen as the reference, and all other structures were aligned to it antigen‐wise using Biotite's structural homolog superposition algorithm. For homooligomeric antigens, the superposition minimizing the distance between the reference and query VHH C_*α*_‐centroids was chosen. Subsequently, interacting VHH‐antigen residues were computed as described above, and an adjacency matrix between sets of epitope residues *E* was generated, where the pairwise distance metric was *d =* 1 – overlap *O*_*ij*_, as defined in Equation (1). Using SciPy 1.15.3., epitope clusters were extracted as the connected components of a sparse graph defined by the epitope adjacency matrix, with connections defined as pairwise distances below 0.5.

(1) $$O_{\mathrm{ij}}=\frac{\left|E_{i}\cap E_{j}\right|}{\left|E_{i}\cup E_{j}\right|}$$

### 4.5 Structural repacking and minimization with Rosetta

A custom RosettaScripts protocol was used to prepare minimized structures of the AVIDbase‐prot subset. The *beta_nov16* energy function with Cartesian weights was used for repacking and minimization, while for scoring the base *beta_nov16* was used (Pavlovicz et al., 2020). All surface residue sidechains (with absolute ASA >40 Å^2^) were repacked, then all side‐chain degrees of freedom were minimized. This was repeated once more, then all sidechains were repacked, followed by all‐atom minimization of all sidechain and backbone atoms in the structure. Any missing densities in the structure were set as jumps. For packing, input sidechain rotamers were considered, and extra rotamers were sampled around the χ_1_ and χ_2_ angles; however, the χ_2_ angle was constrained between 70° and 110° for all aromatic residues. Energy minimization was performed using the nonmonotone L‐BFGS‐Armijo algorithm with a tolerance threshold of 1E‐3. RosettaScripts was also used to compute in silico features for both the final minimized structures and the original crystals. The ΔΔ*G* of binding (*dG_separated*) and change in solvent‐accessible surface area (ΔASA) across the VHH‐antigen interface were computed with the InterfaceAnalyzerMover; the ShapeComplementarity and ContactMolecularSurface (Cao et al., 2022) filters were used to compute their corresponding features, while the BuriedUnsatHbonds filter was used to compute both the number of very buried (*vbuns*) and surface buried (*sbuns*) unsatisfied polar atoms. Interface H‐bonds were counted with HbondMetric, and the nanobody spatial aggregation propensity (SAP) score (Lauer et al., 2012) in absence of antigen was obtained with SapScoreMetric. When comparing the distributions of these descriptors between the crystal structures and Rosetta‐repacked and minimized versions, the Welch *t*‐test was used, and the resulting p‐values were corrected using the Benjamini–Hochberg procedure to control the false discovery rate at *α* = 0.05.

## AUTHOR CONTRIBUTIONS

**Goran Miličić:** Funding acquisition; data curation; methodology; supervision; resources; writing – review and editing. **Jurij Lah:** Funding acquisition; project administration; supervision; writing – review and editing. **Tadej Medved:** Methodology; software; data curation; formal analysis; visualization; writing – original draft. **San Hadži:** Conceptualization; funding acquisition; formal analysis; supervision; writing – review and editing; project administration.

## CONFLICT OF INTEREST STATEMENT

The authors declare no conflict of interest.

## Supporting information


**Table S1:** Detailed descriptions of each column in AVIDbase.
**Table S2:** Composition of AVIDbase standardized structures.
**Supplementary Figure S1:** Analysis of selected structural descriptor distributions in AVIDbase across sequential filtering steps. (a) Violin plots depicting the distribution of change in solvent‐accessible surface area (ΔASA) and average residue temperature B‐factor at the VHH‐antigen interface. Shown are distributions for AVIDbase structures for the following (sequential) filtering steps: before filtering (purple), after removing redundant interfaces within individual PDB structures (intra_redundancy column, orange), after removing cross‐structure redundant interfaces—AVIDbase‐nr (inter_redundancy column, green), after filtering out structures with low interface integrity or low resolution (AVIDbase‐prot, red), and after removing redundant structures from each epitope cluster (epitope_cluster column, gray). (b) Violin plot comparing average interface B‐factor distributions between AVIDbase‐prot (red) and AVIDbase‐low (blue). Statistical significance is determined by the Welch *t*‐test.
**Supplementary Figure S2:** Additional comparisons between in silico properties of Rosetta‐minimized structures and original crystal structures in AVIDbase‐prot. Contains side‐by‐side comparisons of violin plots showing the distribution of selected descriptors for structures that were repacked and minimized with Rosetta (green, left) and the original crystal structures (red, right). Reported *p*‐values were calculated via Welch *t*‐test and corrected for multiple hypothesis testing with the Benjamini–Hochberg procedure at *α* = 0.05.

## Data Availability

The AVIDbase dataset (both structures and data tables) is openly available on Zenodo (doi.org/10.5281/zenodo.20488703). The source code used to prepare the dataset is available on GitHub (github.com/Novartis/AVIDbase).
