# Supplementary material for: AVIDbase: A biologically accurate structural dataset of nanobody‐antigen complexes
Source: Protein Sci. 2026 Aug 24;35(9):e70773. doi: 10.1002/pro.70773 (PMC13501898; doi:10.1002/pro.70773)
Supplement: Supplementary file 1 [file PRO-35-e70773-s001.docx]

Supplementary information

**AVIDbase: a biologically accurate structural dataset of nanobody-antigen complexes**

**Table of Contents**

[Supplementary tables	2](#_Toc236203925)

[Supplementary figures	10](#_Toc236203926)

[Supplementary text	12](#_Toc236203927)

[Definition of interacting partners	12](#_Toc236203928)

[Construction of true biological assembly with symmetry operations	14](#_Toc236203929)

[Annotation of protein, domain and source organisms	14](#_Toc236203930)

[References	15](#_Toc236203931)

# Supplementary tables

**Table S1** – Detailed descriptions of each column in AVIDbase.

| AVIDbase column | Description |
| --- | --- |
| unique_id (index column) | Unique string index corresponding to the interface record. Defined as **“<PDB code>_<interface index>”** for representative records and **“<PDB code>_<interface index>_<variant>”** for redundant ones. |
| PDB | Lowercase 4-letter code corresponding to the PDB crystal structure from which the interface record was obtained. |
| interface_index | Incremented integer index starting at 0. Denotes a unique VHH-antigen interface in the associated PDB structure. |
| variant | Incremented single-letter identifier starting at B. Denotes redundant copies of a unique VHH-antigen interface belonging to a single PDB structure. Not defined for representative interface records. |
| intra_redundancy | **unique_id** pointing to the representative interface record in the associated PDB structure. Determined based on **vhh_pdbx_entity_id**. Defined only for redundant copies of a unique VHH-antigen interface within a single PDB structure. Combine with **inter_redundancy** to filter all redundant VHH-antigen interfaces. |
| inter_redundancy | **unique_id** pointing to the representative interface record across multiple PDB structures. Determined based on CDR1+CDR2+CDR3 sequence identity, **ag_name** identity, and **ag_source_organism** identity. Defined only for redundant copies of a unique VHH-antigen interface across multiple structures. Combine with **intra_redundancy** to filter all redundant VHH-antigen interfaces. |
| interface_integrity | Classification of interface integrity – **high**, **medium** or **low** (see main text Methods). |
| epitope_cluster | Classification of epitope cluster based on structural alignment (see main text Methods). Format: **»<ag_source_organism>/<ag_name>/cluster<cluster index>«**. Cluster index is an incremented integer starting at 1. May be used as an optional layer of redundancy. |
| bio_assembly_id | Integer index corresponding to the biological assembly (as annotated in the PDB) from which the interface record and resulting AVIDbase structures were extracted. »1« corresponds to biological assembly 1 etc. »0« denotes interface records that weren't extracted from any biological assembly, and were instead determined from either the asymmetric unit or constructed from symmetry mates. |
| assembly_strategy | Classification of strategy used to obtain the true biological assembly (**bio_assembly –** extraction of predefined PDB biological assembly, **asym** – extraction from asymmetric unit, **from_symmetry** – reconstruction via symmetry operations). |
| vhh_pdbx_entity_id | Integer index corresponding to the PDBx entity ID of the VHH. Extracted from the mmCIF header of the source PDB structure. |
| vhh_auth_chain_id | Comma-separated list of author-defined chain IDs defining the VHH molecule in the given interface. Format is **»<author chain ID>_<symmetry index>«**, where the symmetry index is defined by Biotite to differentiate copies of identical chains (relevant when **assembly_strategy** is **bio_assembly** or **from_symmetry**). Defines the true biological assembly together with **ag_auth_chain_id**. |
| ag_auth_chain_id | Comma-separated list of author-defined chain IDs defining the antigen in the given interface. Format is **»<author chain ID>_<symmetry index>«**, where the symmetry index is defined by Biotite to differentiate copies of identical chains (relevant when **assembly_strategy** is **bio_assembly** or **from_symmetry**). IDs are sorted alphabetically and with increasing **symmetry index**. Defines the true biological assembly together with **vhh_auth_chain_id**. |
| vhh_chain_id | Comma-separated list of single-letter chain IDs defining the VHH molecule in the corresponding AVIDbase structure. Order is the same as in **vhh_auth_chain_id**. |
| ag_chain_id | Comma-separated list of single-letter chain IDs defining the antigen molecule in the corresponding AVIDbase structure. Order is the same as in **ag_auth_chain_id**. |
| vhh_source_organism | Classification of source organism for the VHH molecule. Format is **“<genus>-<species>”**. |
| ag_name | Classification of antigen name of the format **»<ag_protein>_<ag_domain>«**. If entire protein unit is resolved in the structure, **ag_domain** is omitted. For oligomeric complexes, classification is given as that oligomeric complex's conventional name if applicable, otherwise as a »+«-separated list of **»<ag_protein>_<ag_domain>«** (custom order, priority is given to molecules in contact with VHH). |
| ag_protein | Classification of antigen protein names. Given as a comma-separated list in the same order as in ag_name. |
| ag_domain | Classification of antigen protein domains. Given as a comma-separated list in the same order as in ag_name. |
| ag_source_organism | Classification of source organism for the antigen molecule(s). Format is **“<genus>-<species>”**. Exception is made in the case of viruses, where individual strains are reported as a dash-separated string. For oligomeric antigens, if the source organism is the same for all units, a single classification is reported, otherwise a comma-separated list of classifications in the order of **ag_auth_chain_id** is given. |
| ag_source_organism_subtype | Classification of source organism subtype (serotype, strain, etc.) for the antigen molecule(s). Given as a dash-separated string. For oligomeric antigens, if the source organism is the same for all units, a single classification is reported, otherwise a comma-separated list of classifications in the order of **ag_auth_chain_id** is given. |
| ag_quaternary_structure | Classification of antigen quaternary structure. Format is »monomer« for monomeric antigens, otherwise **»<homo/hetero>-<no. units>-mer«**. |
| resolution | Crystallographic resolution of the associated PDB crystal structure. |
| contains_missing_density | Binary classification of the presence of missing density segments in the structure. |
| vhh_missing_density_spans | Comma-separated list of missing density spans in the VHH structure, defined by residues immediately preceding and following the missing density. Format is **»<preceding residue chain ID>.<preceding residue ID>-<following residue chain ID>.<following residue ID>«**. Chain IDs and residue numbering are as in the AVIDbase processed structures. **Undefined for structures with no missing density in the VHH.** |
| ag_missing_density_spans | Comma-separated list of missing density spans in the antigen structure, defined by residues immediately preceding and following the missing density. Format is **»<preceding residue chain ID>.<preceding residue ID>-<following residue chain ID>.<following residue ID>«**. Chain IDs and residue numbering are as in the AVIDbase processed structures. **Undefined for structures with no missing density in the antigen.** |
| closest_missing_density_span | Classification of missing density span closest to the opposite side of the interface, defined by residues immediately preceding and following the missing density. Format is **»<preceding residue chain ID>.<preceding residue ID>-<following residue chain ID>.<following residue ID>«**. Chain IDs and residue numbering are as in the AVIDbase processed structures. |
| closest_missing_density_distance | Euclidean distance between the closest missing density span and the opposite side of the interface, defined as the shortest distance of the C_α_-atoms brakceting the missing density span from any heavy atom on the opposite side of the interface (see main text Methods). |
| closest_glycan_residue | Classification of closest glycan residue to the opposite side of the interface. Format is **»<glycan chain ID>.<glycan residue ID>-< glycan residue 3-letter code>«**. Chain IDs and residue numbering are as in the AVIDbase processed structures. |
| closest_glycan_distance | Euclidean distance between the closest glycan residue and the opposite side of the interface, defined as the shortest pairwise heavy-atom distance (see main text Methods). |
| expected_cofactors | Comma-separated list of expected cofactors in the structure based on manual assessment of associated UniProt entries, literature and PDB structure. Expected cofactors are given as 3-letter residue codes. |
| closest_cofactor | Classification of closest cofactor to the opposite side of the interface. Format is **»<cofactor chain ID>.< cofactor residue ID>-<cofactor 3-letter code>«**. Chain IDs and residue numbering are as in the AVIDbase processed structures. |
| closest_cofactor_distance | Euclidean distance between the closest cofactor and the opposite side of the interface, defined as the shortest pairwise heavy-atom distance (see main text Methods). |
| noncanonical_residues | Comma-separated list of noncanonical residues species found in the structure (given as 3-letter codes). Includes posttranslational modifications such as phosporylation, methylation, etc. |
| interface_noncanonical_residues | Comma-separated list of noncanonical residues found at the interface. Format is **»<residue chain ID>.<residue ID>-<residue 3-letter code>«**. Chain IDs and residue numbering are as in the AVIDbase processed structures. |
| contains_crystal_waters | Binary classification of presence of cocrystallized waters in the source PDB structure. |
| CDR1 | Amino acid sequence of defined VHH CDR1 – IMGT residues 27-38. |
| CDR2 | Amino acid sequence of defined VHH CDR2 – IMGT residues 56-67, see (Zavrtanik et al. 2018). |
| CDR3 | Amino acid sequence of defined VHH CDR3 – IMGT residues 105-117, see (Zavrtanik et al. 2018). |
| vhh_sequence | Full amino acid sequence of defined VHH molecule. Extracted from the mmCIF header of the associated PDB structure. Expression tags are removed. |
| ag_sequence | Comma-separated list of full amino acid sequences of defined antigen molecules. Extracted from the mmCIF header of the associated PDB structure. Expression tags are removed. Noncanonical residues that aren't posttranslational modifications of canonical amino acids are noted as »X«, otherwise the canonical 1-letter code is used. Order of individual sequences is the same as in **ag_auth_chain_id**. |
| interface_residues | Comma-separated list of interface residues. For explicit definition see main text Methods and (Zavrtanik et al. 2018). Format: **»<residue chain ID>.<residue ID><residue insertion code>«**. Chain IDs and residue numbering are as in the AVIDbase processed structures. |
| vhh_pdb_title | Free text definition of the VHH entity name as defined by the PDB structure's depositing authors. |
| ag_pdb_title | Comma-separated free text definition of the antigen entity names as defined by the PDB structure's depositing authors. Order is the same as in **ag_auth_chain_id**. |
| pdb_structure_title | Free text title of deposited PDB structure corresponding to the interface record. |
| release_date | Release date of deposited crystal structure (format YYYY-MM-DD). |
| pmid | PubMed ID of associated publication. |
| publication_doi | DOI of associated publication. |
| ag_uniprot_name | Comma-separated list of Uniprot entry codes for defined antigen molecules. All entry codes were extracted from the mmCIF header of the associated PDB structure. Order is the same as in **ag_auth_chain_id**. |
| ag_uniprot_accession_code | Comma-separated list of Uniprot accession codes for defined antigen molecules. All entry codes were extracted from the mmCIF header of the associated PDB structure. Order is the same as in **ag_auth_chain_id**. |
| edit_comment | Free-text comment by AVIDbase authors explaining decision taken when defining interface records (where applicable). |
| pymol_interface_selector | Convenience command to highlight interface residues for AVIDbase standardized structures in PyMOL. |

**Table S2** – Composition of AVIDbase standardized structures.

| Entity | Chain ID | Residue numbering | Modifications |
| --- | --- | --- | --- |
| VHH | **H** (reserved) | **IMGT** | N- and C-terminal tags removed (ANARCII)  Se-Met replaced with Met  Repacked and minimized in **AVIDbase-prot-repacked-min** |
| antigen | **uppercase alphabetical** (A,B,C,...) | Identical to source PDB structure (retains insertion codes) | N- and C-terminal tags removed  Se-Met replaced with Met  Repacked and minimized in **AVIDbase-prot-repacked-min** |
| glycans | **lowercase alphabetical** (a,b,c,...) – corresponds to covalently bound protein chain (e. g. **a** if bound to chain **A**) | Renumbered with continuous residue IDs starting at 1 (based on order in the original PDB).  Each oligosaccharide has a single residue ID, monosaccharide units are distinguished by insertion codes. | Removed in **AVIDbase-prot** |
| cofactors | **X** (reserved) | Renumbered with continuous residue IDs starting at 1 (based on order in the original PDB). | Removed in **AVIDbase-prot** |

# Supplementary figures

**Supplementary Figure S1 – Analysis of selected structural descriptor distributions in AVIDbase across sequential filtering steps. a.** Violin plots depicting the distribution of change in solvent-accessible surface area (ΔASA) and average residue temperature B-factor at the VHH-antigen interface. Shown are distributions for AVIDbase structures for the following (sequential) filtering steps: before filtering (purple), after removing redundant interfaces within individual PDB structures (**intra_redundancy** column, orange), after removing cross-structure redundant interfaces – **AVIDbase-nr** (**inter_redundancy** column, green), after filtering out structures with low interface integrity or low resolution (**AVIDbase-prot**, red), and after removing redundant structures from each epitope cluster (**epitope_cluster** column, grey). **b.** Violin plot comparing average interface B-factor distributions between **AVIDbase-prot** (red) and **AVIDbase-low** (blue). Statistical significance is determined by the Welch t-test.

**Supplementary Figure S2 – Additional comparisons between *in silico* properties of Rosetta-minimized structures and original crystal structures in AVIDbase-prot.** Contains side-by-side comparisons of violin plots showing the distribution of selected descriptors for structures that were repacked and minimized with Rosetta (green, left) and the original crystal structures (red, right). Reported p-values were calculated via Welch t-test and corrected for multiple hypothesis testing with the Benjamini-Hochberg procedure at α = 0.05.

# Supplementary text

## Definition of interacting partners

During initial preparation of the dataset, interface records were generated automatically from the most informative assemblies as determined for each PDB structure. First, polypeptide sequences were extracted from the mmCIF header and sorted by ANARCII (Greenshields-Watson et al. 2025) – chains with a sequence score ≥ 24 were labelled as VHH, and the rest as antigens. The information content (number of VHH and antigen molecules in the asymmetric unit, antigen oligomeric state) between the asymmetric unit and the PDB-defined biological assemblies was then compared. If the number of classified VHH molecules was greater in the asymmetric unit and there was only one annotated biological assembly, interface records were prepared from the asymmetric unit, otherwise they were prepared from all defined biological assemblies with at least 1 VHH and 1 non-VHH polypeptide molecule. Each final interface record contained only 1 VHH chain ID.

The interacting molecules in each entry are defined by the **auth_ag_chain_id** (antigen) and **auth_vhh_chain_id** (nanobody) columns. The **auth_ag_chain_id** and **auth_vhh_chain_id** columns contain comma-separated lists of identifiers formatted as **»<author chain ID>_<symmetry index>«**, where the author chain ID is the PDB chain ID as defined by the depositing authors, and the symmetry index is assigned by Biotite to differentiate duplicate chains in a biological assembly. Each interface was then manually reviewed to separate biological interfaces from crystallographic ones (based on calculation of CDR heavy-atom contacts with Euclidean distance ≤ 5 Å, as well as visual inspection of the deposited structure and review of the accompanying publication and UniProt entry), and the definitions of **auth_ag_chain_id** and **auth_vhh_chain_id** were modified where applicable (reason for modification is given in the **edit_comment** column). Assembly information from UniProt was obtained under the **Interaction > Subunit** subsection. All interface records containing crystallographic interfaces or misclassified VHH molecules were removed from consideration. The strategy to obtain each assembly (in the form of AVIDbase standardized structure) was defined in the **assembly_strategy** column (*»asym«* – asymmetric unit, *»bio_assembly«* – biological assembly, *»from_symmetry«* – custom-built structure using symmetry operations). This strategy was manually modified in cases of incorrect or incomplete biological assemblies (either to *»asym«* or *»from_symmetry«*). Assembly with symmetry operations could only be chosen manually, for cases where a homooligomeric antigen was expected, but not found in the predefined biological assemblies. In these cases, the expected antigen oligomerization state and explicit chain IDs needed to be defined manually in **auth_ag_chain_id**.

Expected cofactors in each structure were first automatically added (column **expected_cofactors**) as all non-solvent, non-carbohydrate molecules identified in the source PDB. This list was then manually filtered in each entry to retain only biologically relevant cofactors and remove crystallization agents and other excipients based on literature and UniProt information (e. g. Zn^2+^ ions for zinc finger domains or heme for hemoglobin). Information from UniProt was obtained under the **Function > Cofactor** subsection.

The above information is collected in an Excel table (**assembly_annotation_MANUAL.xlsx**) that was used as input for the generation of both the final AVIDbase dataset and associated standardized structures**.** The table may also serve as a template for further extension of the dataset; columns for user input are highlighted.

## Construction of true biological assembly with symmetry operations

Structures that needed to be built from symmetry were prepared using the following protocol. The unit cell was generated from the asymmetric unit, then duplicated in each direction for a total of 27 cells. Iterating over the exact order of chains manually specified in **auth_ag_chain_id**, the specified chain was isolated, along with all neighboring polypeptide chains within a 5 Å distance. Then, the ΔASA between the selected chain and each individual neighboring chain was computed in isolation, and neighboring chains were ranked by descending ΔASA. From the ranked chains, the one with both the highest buried surface area and appropriate chain ID (next in the **auth_ag_chain_id** list) was selected as part of the true biological assembly. The selected chain was then isolated along with all neighboring chains and the process was repeated until the number of selected chains was equal to the length of the **auth_ag_chain_id** list.

## Annotation of protein, domain and source organisms

AVIDbase contains a custom annotation scheme for antigen names as well as the species and subspecies of VHH and antigen source organisms. The basis for this is information taken from the source PDB structure's mmCIF header, mapped onto each unique molecule based on its *entity.id*. The **ag_name** column contains a single-string identifier combining the name of the antigen at the level of quaternary structure and the specific domain that is resolved in the structure, in the format **“[protein-name]_[protein-domain]”** (e. g. “spike_RBD” for the receptor-binding domain of the *Coronaviridae* spike protein). The domain part of the identifier is omitted if the entire structure is resolved, such as in the case of lysozyme (whose corresponding **ag_name** is »lysozyme«). For heterooligomeric antigens, if the complex has a known unique identifier in the literature, that name is used preferentially, otherwise the name-domain identifiers of the individual subunits are given as a comma-separated list.

Source organism names are collected from data categories *entity_src_gen*, *entity_src_nat* and *pdbx_entity_src_syn* and harmonized based on a custom mapping function. In some cases, expression host organism was incorrectly labeled as the source organism (e. g. *E. coli* listed as the source organism for a *L. glama* nanobody) – these cases were corrected manually based on data from the accompanying publication. The **vhh_source_organism** and **ag_source_organism** columns denote the species of the source organism as **“[genus]-[species]”**, with an exception made for viruses, which are, due to high genetic variability (Sanjuán & Domingo-Calap, 2021), separated on the level of individual strains and annotated by their conventional names (e. g. “sars-cov-2”). In the latter case, serotype/variant/isolate metadata (where available) is reported in the **ag_source_organism_subtype** column for additional information only.

# References

Greenshields-Watson A, Agarwal P, Robinson SA, Williams BH, Gordon GL, Capel HL, et al. (2025) ANARCII: A Generalised Language Model for Antigen Receptor Numbering. BioRxiv 2025.04.16.648720.

Sanjuán R, Domingo-Calap P. Genetic Diversity and Evolution of Viral Populations. (2021) Encyclopedia of Virology 1–5:53–61.

Zavrtanik U, Lukan J, Loris R, Lah J, Hadži S (2018) Structural Basis of Epitope Recognition by Heavy-Chain Camelid Antibodies. J Mol Biol 430(21):4369–4386.
